# Assessment of the Stability of the Palatal Rugae in a 3D-3D Superimposition Technique Following Slow Maxillary Expansion (SME)

**DOI:** 10.1038/s41598-020-59637-5

**Published:** 2020-02-14

**Authors:** Valentina Lanteri, Gianguido Cossellu, Marco Farronato, Alessandro Ugolini, Rosalia Leonardi, Francesca Rusconi, Stefano De Luca, Roberto Biagi, Cinzia Maspero

**Affiliations:** 1 Department of Biomedical Surgical and Dental Sciences, University of Milan, Milan, Italy; 2 Department of Orthondontics, University of Genoa, Genoa, Italy; 3 Department of Orthodontics, University of Catania, Catania, Italy; 4 Área de Identificación Forense, Unidad de Derechos Humanos, Servicio Médico Legal, Santiago de Chile, Chile; 5 AgEstimation Project, Macerata, Italy; 6 Fondazione IRCCS Ca’ Granda, Ospedale Maggiore Policlinico, Milan, Italy

**Keywords:** Bioinformatics, Bioinformatics, Diagnostic markers, Diagnostic markers

## Abstract

The Palatal Rugae are considered a useful human identification marker for both orthodontists and forensic personnel. The principal aim of the present study was to evaluate the stability of palatal rugae with a 3D-3D superimposition procedure following Slow Maxillary Expansion (SME), in order to assess whether they kept their uniqueness and validity for human identification, even after a specific dental treatment. For this purpose, a sample of 27 digital dental models - belonging to growing patients (13 males and 14 females), aged between 8.5 and 15 years, who underwent SME therapy - was retrospectively studied and compared with a control group of 27 untreated subjects - (13 males and 14 females). Digital dental models were obtained pre-treatment and at device removal; both were processed by means of an intraoral scanner. A superimposition procedure was thus performed to reach the minimum point-to-point distance between two models of palatal rugae. Intra- and inter-observer differences were statistically analyzed by paired Wilcoxon test and Intra-class Correlation coefficient (ICC), showing values larger than 0.93. There was no difference in Root-Mean-Square (RMS) values between untreated control subjects and subjects treated with Leaf Expander (p = 0.062). A RMS value of 0.43 was the threshold to distinguish the pooled group (“Untreated” and “Leaf”) from any mismatch. According to the obtained results, this study failed to reject the null hypothesis and presented no differences between the RMS values of the Test group and the RMS values of the untreated control group. This work highlighted the usefulness of 3D superimposition procedure for purposes of human identification, in subjects undergoing dental treatment. However, keeping in sight the forensic use of this technique as a helpful probation element in court, further studies should be performed to confirm these findings.

## Introduction

The analysis of Palatal Rugae, also known as *Plicae Palatinae Transversae* and *Rugae Palatinae*, is one of the most reliable methods for human identification, mostly in cases of corpses that lack valuable dental information during major disasters^1–3^. Due to its uniqueness, stability throughout the whole life and their resistance to diseases and several extraoral environmental damages - such as the action of high temperatures and maceration- the palatal rugae have been widely studied in the last decades in order to verify their validity as an identification marker^4–6^.

In the last decades, few researches have been carried out to analyze the changes in the palatal rugae morphology submitted to some orthodontic treatments such as, for example, the Palatal Expansion^7–9^. Applying this type of orthopedic forces to the maxillary arch to expand itself in growing subjects during the correction of malocclusions, could cause significant morphological changes to the rugae^10–13^. There are many types of maxillary expansion methods and various recommended expansion rates, which can result in Rapid Maxillary Expansion (RME hereafter)^12,13^ or Slow Maxillary Expansion (SME hereafter)^14^. According to the literature^15^, in case of SME, skeletal transversal width is present but reported to be lower than dento-alveolar expansion. Unfortunately, evidence on changes of the palatal rugae following SME is inconclusive^16,17^.

In children, growth is expected to occur in the rugae region, affecting both palatal depth and surface area. Therefore, verifying the palatal rugae stability in growing subjects could be even more challenging^18–20^.

In this study, the effects of SME on the palatal rugae patterns and stability were assessed in a sample of 3D digital dental models of Italian growing subjects for the first time. The advantages of digital study models include easy and fast storage, transfer of data, immediate access and virtual simulation^21,22^. In addition, the accuracy of direct digital models added the plug-and-play capability of an automatic exchange of information^21^.

Although the validity of these models has been widely studied for human identification, a method for superimposing pre- and post-treatment digital dental models is still under evaluation. Some previous studies showed that the medial 2/3 of the third rugae, and the regional palatal vault dorsal to the third rugae, are stable regions that can be used for 3D digital model superimposition^23,24^. In another research^25^, the morphological characteristics of the palatal rugae were examined in a Korean sample of 343 adult subjects. The results indicated that the numbers of the palatal rugae varied greatly among individuals and this could affect the antero-posterior position of the third primary ruga. More recently, a metrical assessment of point-to-point differences between two three-dimensional (3D) models of the upper dental arch was used to test the discriminating potential of 3D image of palatal rugae^26^. The authors showed that the palatal rugae are unique and stable, and that 3D image acquisition systems could contribute to the morphological and metrical assessment of these particular structures. Finally, the “Identity Base” (IB) system was introduced to develop a complex information system and a personal identification protocol by means of three-dimensional palatal scans in digital format^27^.

The present study aimed to evaluate the stability of palatal rugae with a 3D-3D superimposition procedure following SME to determine if they can be used as an appropriate reference area for human identification in forensic context. Thus, the null hypothesis (H0) presented no differences between the RMS values of the Test group and the RMS values of the untreated control group needed to be assessed.

## Material and Methods

### Sample

A retrospective study was carried out, in which digital dental casts of orthodontically treated growing patients were evaluated at two different intervals: before and after treatment. The subjects sample were collected from the database of the UOC Chirurgia Maxillo Facciale e Odontostomatologia - Fondazione IRCCS Ca’ Granda, Ospedale Maggiore Policlinico - Department of Biomedical Surgical and Dental Sciences University of Milan (Italy). Informed consent was obtained, before dental treatment, from the parents and/or legal guardians of all subjects who were younger than 18 years for study participation.

Digital dental models were obtained pre-treatment and at device removal, and processed by means of an intraoral scanner: 3Shape D250 (3Shape, Copenhagen, Denmark). The validity and precision of the scanner are both of ±0.05 mm.

Subjects were selected according to the following eligible criteria: Early or mid-mixed dentition, with both primary and second molars (E + E hereafter) preserved, Cervical Vertebral Stage 1 through 3 (CVS methods 1–3), Angle Class I or Class II malocclusion, no previous orthodontic treatments and maxillary arch constriction. The exclusion criteria were as follows: The presence of craniofacial abnormalities, previous extraction or surgical treatment, Angle Class III malocclusion, Temporomandibular Joint (TMJ hereafter) dysfunctions or caries of E. Test group consisted of 27 subjects (13 males - 14 females), mean age of 8.5 ± 1.5 years old, who underwent SME (Leaf Expander) therapy. The devices were banded by the same operator on the primary second molars.

The expanders presented the same type of screw (6 mm – 450 g) with an anterior arm up to the canines; no subsequent orthodontic treatment was to either the maxilla nor the mandible. The expander was activated following the protocol previously described^24^. All the expanders were removed after a period of 9 to 11 months. The specific amount of expansion in each single case was not evaluated, although the expansion was clinically achieved in all the subjects treated.

The Control group consisted of 27 subjects with maxillary arch constriction (13 males and 14 females), mean age of 8.5 ± 1.5 years, and whom had not received any treatment for at least 10 months. The control selection criterion was the same to the criteria of the experimental group selection.

Among all the possible combinations, 35 mismatches were randomly selected in order to compare the results with the matching groups (untreated Control and Leaf). The “Mismatches” group is represented by the overlap of different subjects.

### Superimposition

Only one superimposition procedure was performed to study the differences between the pre and post-treatment models in the palatal rugae area. According to literature^24^, this area is relatively stable during an individuals’ whole life.

**Figure 1:**
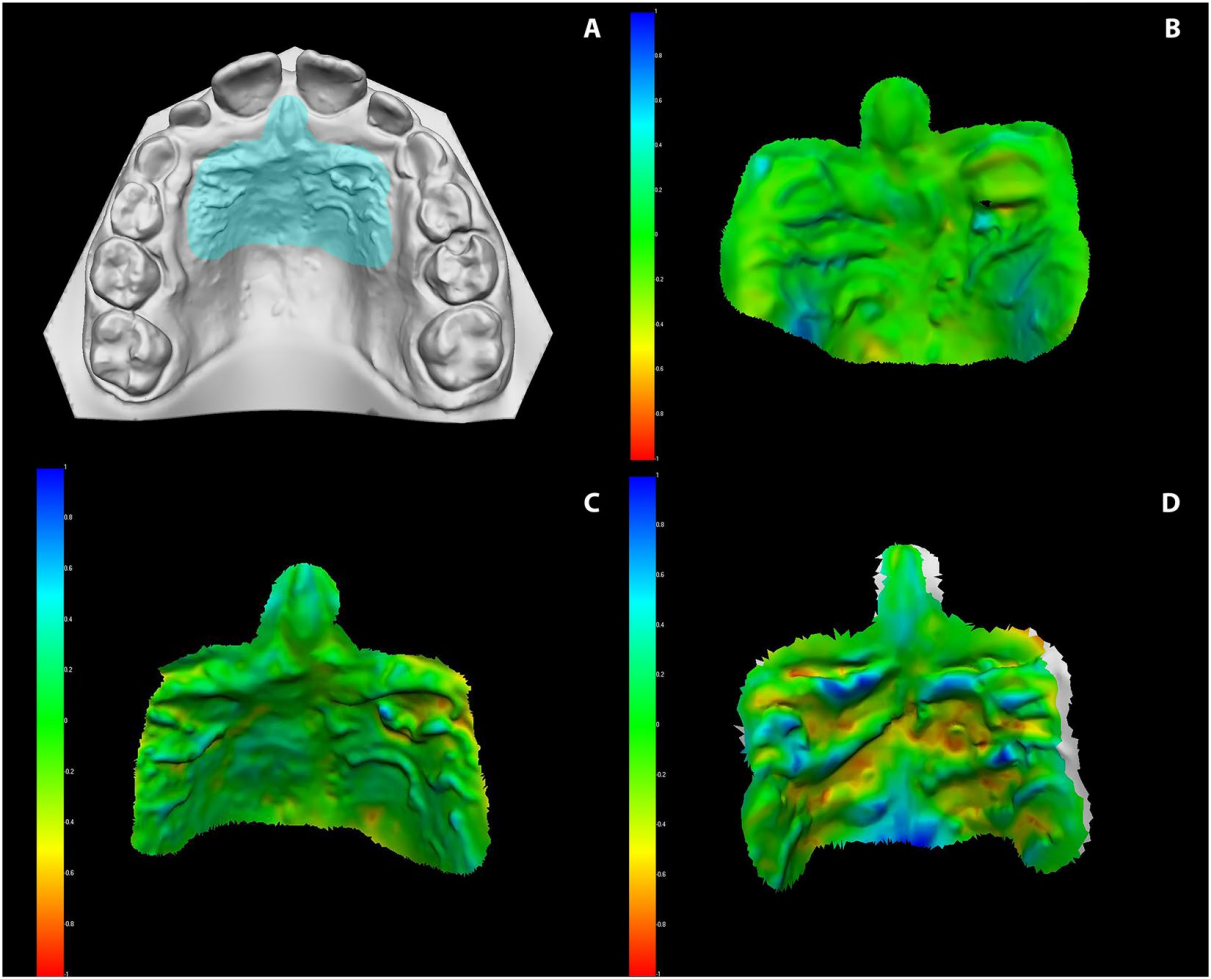
(**A**) Example of a 3D-scanned digital model of the palatal area; (**B**) Chromatic representation of differences between two superimposed models in “Untreated Control” subjects; (**C**) Chromatic representation of differences between two superimposed models in “Leaf” subjects before and after SME; (**D**) Chromatic representation of differences between two superimposed models in case of mismatches. Green means perfect match whereas yellow and red mean areas of discordances between the two digital models.

During the first step, the palatal area including the rugae were manually selected and then isolated from both pre and post-treatment models (Fig. 1A). Then, the models of the two obtained palatal areas were automatically superimposed by VAM software (VECTRA Analysis Module, version 3.7.6, Fairfield, New Jersey), through a best-fit algorithm, in order to reach the best match between them. In this case, since an automatic best-fit superimposition method was used, fiduciary maxillary stable structures or reference points were not considered.

After the two areas were registered, the software was requested to provide the point-to-point distance between the entire registered surfaces. The superimposition results were illustrated in a difference map, in which discrepancies (in mm) are represented through a color-coded scale: The green meant perfectly matching surface, the red meant test model surface was positively positioned - relative to reference model - and the blue meant test model surface was negatively positioned, relative to reference model.

### Statistical analysis

Sample size was calculated *a priori*, based on a primary outcome Root Mean Square (RMS hereafter), as continuous outcome to obtain a statistical power of the study greater than 0.85, using the mean values and standard deviations of RMS found by Gibelli *et al*.^26^. RMS is the square root of the arithmetic mean of the squares of the point-to-point distance between the areas with an identical coordinate system, while the achieved errors are measured in the same units as the obtained response. Based on these parameters, the sample size required was 25 patients in each group.

To determine the error of method, RMS calculation was repeated 7 days after the first measurement was performed on 15 randomly selected casts, by the same observer (FR) and by a second observer (GC) - two senior orthodontists with varying levels of experience. The individual observer’s analyses were not disclosed to the other observer.

Intra and inter-observer differences were statistically analyzed by paired Wilcoxon test. In addition, the Intra-class Correlation coefficients (ICC) were calculated.

Shapiro-Wilk’s test showed that RMS data were normally distributed, therefore parametric statistics were thus applied. Descriptive statistics, median value, standard deviation and 95% Confidence Interval (CI) were calculated. Differences between groups were computed with an ANOVA test and post-hoc Bonferroni tests. Probabilities of less than 0.05 were accepted as significant in all statistical analyses (except for the Bonferroni test for which the accepted probability was 0.017). All statistical analyses were performed using IBM SPSS Statistics ver. 21.0 software (IBM, Armonk, NY, USA), with significance level set at p < 0.05.

### Compliance with Ethical standards

This study has been approved by the Local Ethic Committee, *Fondazione IRCCS Ospedale Maggiore Policlinico*, Milan, Italy (Prot. n° 421, 09/03/2016). The study was carried out in accordance with the ethical standards laid down by the Declaration of Helsinki (Finland).

## Results

The ICC values for the intra- and inter-observer agreement were 0.955 (95% CI: 0.931–0.980; p < 0.001) and 0.936 (95% CI: 0.918–0.975; p < 0.001). Overall, the method error was considered negligible.

Most of the superimposed surfaces were green, which indicates that the reference model and the test model had corresponded with each other. Cross-bites and traversal maxillary discrepancies were corrected at the end of the active expansion phase in 100% of the subjects treated with the Leaf expander. The RMS threshold was found to be statistically different in the three analyzed groups. Two subjects for each group were excluded due to inaccurate scans in the palatal area. The final sample consisted of 25 subjects for the test group (12 boys and 13 girls) and 25 subjects for the control group (13 boys and 12 girls).

**Figure 2:**
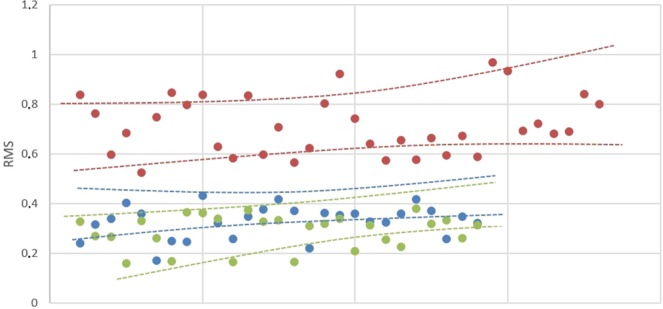
Graphic representation of the RMS values dispersion, according to the different group of subjects: Mismatch (red circles); Leaf (blue circles); Untreated Control (green circles).

Subjects in the “Untreated Control” group reported an average RMS of 0.28 (95% CI 0.24–0.35); the “Leaf” group reported a comparable value to the normal RMS group of 0.34 (95% CI 0.31–0.43). These two groups represented the overlap of the same subject and there was no difference in mean RMS values between untreated subjects and those treated with Leaf Expander (p = 0.062). The “Mismatches” group is represented by the overlap of different subjects and the RMS value is statistically higher (0.71, 95% CI 0.58–0.81) compared to the “Untreated Control” group (p < 0.001) and “Leaf” (p < 0.001). In no case the values of the groups “Untreated Control” and “Leaf” were superimposed with those of the “Mismatches” group (Fig. 1B–D, and Table 1): RMS value of 0.43 was the threshold to distinguish the pooled group (“Untreated Control” and “Leaf”) from the Mismatches (Fig. 2).

**Table 1:** RMS values according to the different model.

RMS			
	Leaf	Mismatches	Untreated
Mean	0.34	0.71	0.28
SD	0.08	0.12	0.14
CI 95%	0.31	0.58	0.24
	0.43	0.81	0.35
Post-hoc	<0.001	<0.001	0.062
	Leaf vs Mism	Mism vs Norm	Leaf vs Norm

SD = Standard Deviation; CI = Confidence Interval; Mismatch = Overlap of different subjects.

## Discussion

### Method

Rugae patterns had previously been studied as anatomical structures and identification markers in several cases: between different subjects and ethnicity, in cases of edentulous subjects and for subjects following orthodontic treatment (expansion or extractions). Different methodologies were also applied to evaluate their anatomical uniqueness and overall stability: intraoral inspection, impressions, plaster casts, digital models, digital photography and stereophotogrammetry^28–30^. 3D models have been proven to be an effective tool and as an alternative method for evaluating palatal rugae patterns in human identification^28–30^. In addition, superimposing 3D digital models is a practical, user-friendly and relatively quick and inexpensive method that could be suitable for specific forensic scenarios, in which identification of an individual is needed.

This study is the first to thoroughly test the effect of SME on superimposition of serial 3D digital dental models obtained by using intraoral scans in a sample of growing subjects aged between 8.5 and 15 years, submitted to this kind of treatment.

Best-fit Superimposition Procedure (i.e. the search for maximum correspondence between two models) is more precise and requires a greater calculation effort of the computer since the procedure is repeated until the difference between the surfaces is minimized. In this case, the RMS and SD values of the distance between the two models indicate how accurate the process is, once the images have been superimposed on the centroid.

It has been widely demonstrated that intraoral scanning may be more accurate, compared to digital models generated by desktop-scanning of conventional impression/plaster models^31^. In fact, direct digitization by means of the intraoral scanner had less systematic errors than the physical model fabrication step with a milling machine from an intraoral scan. In general, the main advantages of intraoral scanners are the following: (1) increased adoption in clinical practices worldwide; (2) different models of portable cart-free systems with a single USB cable - that can be plugged into any workstation - can be developed; (3) improved accuracy of the directly obtained digital models; (4) lost devices could easily be refabricated using the digital files from a database in the “Cloud”^21^; (5) they consume less time and reduce patient exposure to unnecessary radiation, thus being very useful in forensic contexts.

### Patients

Patients younger than 15 years were recruited because of the growth activity of the palatal suture had been reported to reduce around 14 years of age^32^. In fact, the optimal age for SME or RME would be below 13 to 15 years of age, when growth of the mid-palatal suture typically have ceased^11,12^.

To date, although possible modifications of the palatal rugae have been widely discussed as a consequence of some dental treatments, they have not been properly treated in the forensic literature^8,33^. According to published studies^8,34,35^, the shape of the palatine rugae remained consistent with different orthodontic treatment modalities and very limited changes have been observed in their orientation. However, based on certain orthodontic procedures related to maxillary expansion and front tooth restoration treatments, length of palatal rugae has been shown to vary such as: significant increases in the third rugae length during RME, or significant reductions in the second and third rugae length after the extraction treatment.

### Results

The obtained results pointed out that this study failed to reject the Null hypothesis (H0): there were no differences between the RMS values of the Test group and the RMS values of the untreated control group. A small RMS value implied that the estimated mean was close to the true mean, indicating in turn that the estimated SD was close to RMS and the best-fit superposition was accurate and precise (see Table 1). Therefore, this study showed that the use of orthodontic treatments, specifically SME, did not significantly alter the pattern of the palatal rugae in the growing subjects. This is consistent with the findings of previous studies^26,36^. When a comparison is carried out, both the RME and SME were able to produce some changes, but RME generated greater orthopedic changes. In general, the maxillary expansion caused more dento-alveolar and buccal tipping than skeletal expansion^36^.

### Forensics

Eventually, in regards to the intra and inter-observer errors, they were considerably negligible showing that this method is precise and highly reproducible. In the forensic field, reproducible means that, with known probability, different examiners obtain the same result when analyzing the same samples^37^. On the other side, precision refers to the closeness of a value to the real one^35,38^. Therefore, a strong system must be capable of giving consistent measurements, regardless of who is operating it and with what^37,39^.

This study proved that rugoscopy has potential to contribute to human identification, despite certain orthodontic treatment modalities affecting the anteroposterior or transverse dimensions of the palate. This work also highlighted its validity as an additional tool, along with the dentition and genetic analyses, for assessing the identity of an individual in several forensic contexts or when identification by routine methods such as fingerprints analysis is not possible. In fact, the palate is resistant to the action of fire and maceration because of its fibrous structure (keratinized tissue) and anatomically protected position^2^.

It means that the 3D comparison of palatal rugae area can be applied reliably to support positive identifications in court because uniqueness and stability guarantee that a subject examined *postmortem* can be linked exclusively to the same area (from a missing person) examined *antemortem*.

Some limitations should be highlighted when interpreting the data of the present research. Although the palatal rugae area are relatively stable during an individual’s whole life^24^, the sample of this study was mainly limited to Italian growing subjects aged between 8.5 and 15 years, and the morphological changes of palatal rugae area, over a longer period, were not analyzed. Future studies with a longitudinal sample should be performed to evaluate if significant differences are observed in the palatal area because of changes that the palatal mucosa endure over a couple of years to longer term^40^.

Keeping in mind the forensic use of this technique as a helpful probation element in court^2,30,37,39,41^, further studies - using increased number of observers with different levels of experience, larger sample size, different dental treatment such as the Rapid Maxillary Expansion (RME) and various patient groups - are recommended to confirm and generalize the accuracy of these findings. Extended training of an operator is also necessary and should be focused on the quality and quantity of the material available and its impact on the uniqueness of this type of forensic evidence. Finally, various samples from different countries should be considered in order to compare these findings with other populations^42^.

## Conclusions

For the first time, within the limitations of this study, it has been demonstrated that the 3D anatomical morphology of the palatal rugae area is not affected by specific dental treatments, thus indicating that it can maintain its morphological patterns of individuality even after undergoing SME. The superimposition process is highly reproducible and provides, in a simple and inexpensive way, accurate results. The lack of differences statistically significant between the pre- and post-treatment models supports and gives reliability for the forensic applications based on the comparison of the morphology of palatal rugae.

## Data Availability

The datasets generated and/or analysed during the current study are available from the corresponding author, on a reasonable request.
